# Administration of Maresin-1 ameliorates the physiopathology of experimental autoimmune encephalomyelitis

**DOI:** 10.1186/s12974-022-02386-1

**Published:** 2022-02-02

**Authors:** Alba Sánchez-Fernández, Stephanie Zandee, Mauricio Mastrogiovanni, Marc Charabati, Homero Rubbo, Alexandre Prat, Rubèn López-Vales

**Affiliations:** 1 Institut de Neurociencies and Departament de Biologia Cel lular, Fisiologia i Immunologia, Facultat de Medicina, Universitat Autonoma de Barcelona, 08193 Bellaterra, Catalonia Spain; 2 Centro de Investigación Biomédica en Red de Enfermedades Neurodegenerativas (CIBERNED), Barcelona, Spain; 3 Department of Neuroscience, Faculty of Medicine, Université de Montréal and Neuroimmunology Unit, Centre de Recherche du CHUM (CRCHUM), Montréal, Québec Canada; 4 Departamento de Bioquímica, Facultad de Medicina and Centro de Investigaciones Biomédicas (CEINBIO), Universidad de La República, Montevideo, Uruguay

**Keywords:** Experimental autoimmune encephalomyelitis, Inflammation, Maresin-1, Multiple Sclerosis, Resolution of inflammation, Specialized pro-resolving mediators

## Abstract

**Background:**

Resolution of inflammation is an active and regulated process that leads to the clearance of cell debris and immune cells from the challenged tissue, facilitating the recovery of homeostasis. This physiological response is coordinated by endogenous bioactive lipids known as specialized pro-resolving mediators (SPMs). When resolution fails, inflammation becomes uncontrolled leading chronic inflammation and tissue damage, as occurs in multiple sclerosis (MS).

**Methods:**

SPMs and the key biosynthetic enzymes involved in SPM production were analysed by metabololipidomics and qPCR in active brain lesions, serum and peripheral blood mononuclear cells (PBMC) of MS patients as well as in the spinal cord of mice with experimental autoimmune encephalomyelitis (EAE). We also tested the therapeutic actions of the SPM coined Maresin-1 (MaR1) in EAE mice and studied its impact on inflammation by doing luminex and flow cytometry analysis.

**Results:**

We show that levels of MaR1 and other SPMs were below the limit of detection or not increased in the spinal cord of EAE mice, whereas the production of pro-inflammatory eicosanoids was induced during disease progression. Similarly, we reveal that SPMs were undetected in serum and active brain lesion samples of MS patients, which was linked to impaired expression of the enzymes involved in the biosynthetic pathways of SPMs. We demonstrate that exogenous administration of MaR1 in EAE mice suppressed the protein levels of various pro-inflammatory cytokines and reduced immune cells counts in the spinal cord and blood. MaR1 also decreased the numbers of Th1 cells but increased the accumulation of regulatory T cells and drove macrophage polarization towards an anti-inflammatory phenotype. Importantly, we provide clear evidence that administration of MaR1 in mice with clinical signs of EAE enhanced neurological outcomes and protected from demyelination.

**Conclusions:**

This study reveals that there is an imbalance in the production of SPMs in MS patients and in EAE mice, and that increasing the bioavailability of SPMs, such as MaR1, minimizes inflammation and mediates therapeutic actions. Thus, these data suggest that immunoresolvent therapies, such as MaR1, could be a novel avenue for the treatment of MS.

**Supplementary Information:**

The online version contains supplementary material available at 10.1186/s12974-022-02386-1.

## Background

Acute inflammation is a key physiological mechanism that promotes the repair of injured tissues and eliminates infectious organisms and toxic agents. This response is tightly controlled and typically ends with the elimination of the immune cells and cellular debris from the tissue, paving the way for the recovery of homeostasis [1–3]. Contrariwise, uncontrolled inflammation, as it occurs when the resolution phase fails, becomes harmful to the tissue, and it is a hallmark of a wide variety of pathological conditions, including in MS [2, 4–8].

For many years, resolution of inflammation was believed to be a passive process triggered by the dilution of pro-inflammatory mediators at the challenged tissue, thus halting the recruitment of leukocytes from circulation. However, it has now become clear that this process is an active and coordinated event orchestrated by a class of lipids known as SPMs [2, 7]. This bioactive lipid family includes lipoxins (LX), resolvin D series (RvD), resolvin E series, (RvE), protectins (PD/NPD) and maresins (MaR) [2, 7, 9]. In immune cells, SPMs are naturally synthesized from omega-3 and omega-6 polyunsaturated fatty acids (PUFA) through a family of lipoxygenases (i.e., 12- and 15-LOX) [3, 10]. Failure to produce adequate amounts of SPMs has been associated with persistent inflammation in many inflammatory disorders, such as asthma, atherosclerosis, ulcerative colitis, spinal cord injury or Alzheimer’s disease, among others [2, 4, 5, 11, 12]. Despite emerging data revealing that SPMs might control inflammation in central nervous system (CNS) disorders, there are very few studies addressing their importance in neuroinflammatory conditions, such as MS.

MS is a chronic neuroinflammatory and demyelinating disease of the CNS affecting around 2.5 million individuals worldwide [13–15]. Although the exact cause of MS is not yet known, a hallmark of its physiopathology is the presence of persistent numbers of immune cells in the white and grey matter of the CNS [13–16]. Indeed, neuropathological studies show that inflammation is present in all MS stages and that infiltrating leukocytes drive the formation of demyelinated and neurodegenerative lesions [15, 17, 18].

To our knowledge, there are currently few studies addressing the contribution of SPMs in MS patients on animal models [6, 19–21]. In an early report, a connection between disease severity and SPMs production was suggested in a small cohort of MS patients since the authors found increased levels of RvD1 and NPD1 in the cerebrospinal fluid (CSF) of highly active MS patients [6]. A more recent work uncovered that progressive and relapsing–remitting MS patients had increased plasma levels of pro-inflammatory eicosanoids, whereas SPMs were reduced or undetectable [20]. Moreover, this study also found lower transcripts of the SPM biosynthetic enzymes and receptors in leukocytes from MS patients, suggesting an imbalance in peripheral induction of the resolution pathways in MS patients [20]. Interestingly, prophylactic administration of two SPM known as RvD1 and LXA4 has resulted in beneficial actions experimental autoimmune encephalomyelitis (EAE) [19, 21]. However, whether impairment in SPM production occurs in the CNS of MS patients and EAE mice, and whether administration of SPMs that are undetected in blood and CNS samples of MS patients halters inflammation and exerts therapeutic effects in animal models of MS has not been elucidated.

Herein, we reveal that there is impaired production of SPMs in active brain lesions and serum samples of MS patients, as well as in the CNS of mice undergoing EAE. We further connect these results with a defective expression of enzymes involved in the synthesis of SPMs in peripheral blood mononuclear cells (PBMCs) and in brain lesions of MS individuals, as well as, in the spinal cord of EAE mice. We also reveal that the exogenous administration of the DHA-derived SPM coined MaR1 in EAE mice once they showed the clinical signs of the disease modulates different mechanisms of inflammation and confers protection against neurological deficits and demyelination.

## Methods

### MS brain tissue collection

Autopsies on patients from Centre de Recherche du Centre Hospitalier de l'Université de Montreal diagnosed with clinical and neuropathological MS according to the revised 2010 McDonald’s criteria [22] had informed consent as approved by the Centre Hospitalier de l'Université de Montréal ethics committee (BH07.001). Autopsy samples were cryopreserved, and lesions classified using Luxol Fast Blue (LFB)/Haematoxylin & Eosin staining and Oil Red O staining as previously published [23, 24]. For qPCR analysis, normal-appearing white matter (NAWM) and active lesion (AL) representing 7 secondary-progressive MS (SPMS) and 2 relapsing–remitting MS (RRMS) were used. The median age at death was 50 years old (range from 26 to 65 years old) (Additional file 1: Table S1). Regions of interest (lesion or NAWM) for RNA isolation were dissected manually from 5 to 6 50 µm cryosections per block. For lipidomic analysis, normal-appearing white matter (NAWM) and active lesions (AL) representing 6 secondary-progressive MS (SPMS) and 2 relapsing–remitting MS (RRMS) were used. The median age at death was 50 years old (range from 26 to 65 years old) (Additional file 1: Table S2).

### MS and healthy donor PBMC and serum collection

For isolation of the peripheral blood mononuclear cells (PBMCs), blood samples were collected from 8 patients with relapsing–remitting MS (RRMS) and 3 with secondary-progressive MS (SPMS), recruited at the MS Clinic at the Centre de Recherche du Centre Hospitalier de l'Université de Montréal. The mean age was 43 years old (ranged from 27 to 65) (Additional file 1: Table S1). 8 healthy volunteers were included as controls, whose mean age was 35 years old (range from 24 to 42 years old) (Additional file 1: Table S1). PBMCs were isolated from blood samples collected in EDTA-coated Vacutainer tubes (BD Biosciences, Oakville, ON, Canada), using a Ficoll density gradient as previously described [25].

Moreover, serum samples were also obtained from 10 patients with RRMS and 10 healthy volunteers. The mean age was 47 years old (ranging from 36 to 55) and 43 years old (ranging from 35 to 55) in the MS and healthy donor group, respectively (Additional file 1: Table S2).

A written informed consent was obtained from patients and healthy donors in accordance with the local ethics committee (CRCHUM research ethic committee approval number BH07.001).

### Experimental autoimmune encephalomyelitis

All experimental procedures were approved by the Universitat Autònoma de Barcelona Animal Experimentation Ethical Committee (CEEAH 2878) and followed the European Communities Council Directive 2010/63/EU, and the methods were carried out in accordance with the approved guidelines.

Female adult C57Bl/6 (8–10 weeks old; Charles River Laboratories) were sedated with intramuscular injection of a mixture of ketamine (22 mg/kg) (Imalgen 1000, Merial) and xylazine (2.5 mg/kg) (Rompun, Bayer). Experimental autoimmune encephalomyelitis (EAE) was actively induced by subcutaneously immunization with 300 µg of myelin oligodendrocyte glycoprotein peptide 35–55 (MOG_35-55_ MEVGWYRSPFSRVVHLYRNGK, Thermo Fisher Scientific, MA, USA) in 200 µL Complete Freund’s Adjuvant (CFA) (Difco, MI, USA) supplemented with 4 mg/mL of heat inactivated *Mycobacterium tuberculosis* (Difco, MI, USA). Intraperitoneal (i.p.) injections of 400 ng of pertussis toxin (Sigma-Aldrich, ON, USA) in 100 µL sterile saline were administered at the time of induction and again 48 h later. All the mice were housed with food and water ad libitum at a room temperature of 22 ± 2ºC under 12:12 h light–dark cycle. EAE onset was considered on the day animals showed the first signs of disease (EAE score 0.5 or 1; around day 9–10 post-immunization). Disease peak was considered on the day EAE score did not increase from the previous day (day 16–18 post-immunization). Chronic phase of the disease was considered at day 21 post-immunization if EAE score decreased or was maintained with respect to disease peak.

### EAE functional evaluation

Mice were daily scored from day 0 to day 21 after induction of EAE. The researcher was blind to experimental groups during the functional evaluation. A 6-point scale was used to evaluate the clinical signs of EAE: 0 = normal walking, 0.5 = partially paralysed tail, 1 = fully paralysed tail, 2 = mild hind limb weakness, quick righting reflex, 3 = severe hind limb weakness, slow righting reflex, unable to bear weight, 3.5 = severe hind limb weakness, partial paralysis of hind limb, 4 = complete paralysis of at least one hind limb, 4.5 = complete paralysis of one or both hind limbs and trunk weakness, 5 = complete paralysis of one or both hind limbs, forelimb weakness or paralysis, and 6 = moribund.

### Drug administration

EAE-induced mice were randomly assigned to the treatment and control experimental groups. Daily injections (i.p.) of 1 µg of MaR1 (7R,14S-dihydroxy-4Z, 8E, 10E,12Z, 16Z, 19Z-DHA; Cayman Chemical)) in 200 µL of sterile saline (0.9% NaCl) or vehicle were initiated in each individual mouse the first day of the clinical signs until the end of the study 21 days after induction. The dose of MaR1 was chosen based on the effectivity of this SPM in spinal cord injury [4].

### Real-time quantitative PCR assay (qPCR)

Unimmunized and immunized female C57Bl6/J mice at different stages of the disease (onset: 11/12 days after EAE induction, peak: 17 days after EAE induction, and chronic phase: 21 days after EAE induction) were transcardially perfused with 60 mL of sterile saline (0.9% NaCl) and lumbar spinal cords were harvested. Total RNA was isolated using RNeasy Lipid Tissue Mini Kit (Qiagen) according to the manufacturer’s instruction.

RNA was isolated from post-mortem MS brain samples and PBMCs from MS patients and healthy donors using RNeasy Lipid Tissue Micro Kit (Qiagen) and QIAamp RNA Blood Tissue Mini Kit (Qiagen), respectively, according to the manufacturer’s guidelines.

1 µg of RNA isolated from mouse spinal cords and human PBMCs, and 0.1 µg of RNA from human brain samples, was then retrotranscribed using cDNA Reverse Transcription Kit (Applied Biosystems). qPCR was performed using TaqMan reagents from Applied Biosystems and the following TaqMan-designed primers (Thermo Fisher Scientific): mouse *Alox-12/15* (Mm00507789_m1), human *ALOX12* (Hs00167524_m1) and human *ALOX-15* (Hs00993765_g1). Mouse *Gapdh* (Mm99999915_g1) and human *GAPDH* (Hs02786624_g1) were used as housekeeping gene. qPCR was performed using the Bio-Rad CFX384 apparatus and software. The amount of cDNA was calculated based on the threshold cycle (CT) value and was standardized by the amount of *Gapdh* using the 2^−ΔΔCT^ method [26].

### Lipidomic analysis

Lipidomic analysis was done on brain AL and NAWM tissue obtained from MS patients, serum samples collected from MS and healthy volunteers and spinal cord samples harvested from both naïve and EAE mice at different stages of the disease (onset, peak and chronic phase).

All samples were thawed and weighted and 180 µL MeOH containing internal deuterated standard mix was added. Homogenization was performed with zirconium oxide beads in a bullet blender (Next Advance) according to the manufacturer’s instructions and further centrifugation at 14.000 g was done. Supernatants were diluted with 0.1% formic acid (1:9) and oxylipin analysis was performed. Human serum samples (500 µL) were thawed and diluted in equal volume of 20% MeOH containing internal deuterated standard mix. After centrifugation at 14.000 g, supernatants were submitted to oxylipin analysis as described below.

Oxylipin analysis was performed as previously described [27]. Briefly, samples are transferred to pre-conditioned Strata™-X SPE cartridges (Phenomenex, Torrance, CA) and washed with 10% MeOH solution in acidified water (HCl, pH 3). Then oxylipins were eluted with MeOH under vacuum, dried and reconstituted with 50% MeOH. Extracts were separated on a Luna C18(2) column (Phenomenex, Torrance, CA) and analysed by negative mode electrospray ionization using scheduled multiple reaction monitoring (sMRM) on a spectrometer QTRAP4500 (ABSciex, Framingham, MA). Identification and quantitation assessed by internal standard methods were performed with Analyst 1.6.2 software (ABSciex).

### Histological analysis

EAE mice were euthanized on day 21 post-immunization with an overdose of pentobarbital sodium (Dolethal) and transcardially perfused with 4% paraformaldehyde (PFA) in 0.1 M phosphate buffer (PB). Lumbar segments of spinal cords were harvested, post-fixed in 4% PFA for 2 h and cryoprotected in 30% sucrose in 0.1 M at 4 °C for at least 48 h. Spinal cords were embedded in TissueTek OCT (Sakura), cut into transversal Section (15 µm thick) with a cryostat (Leica) between L3 and L5 segments and serially picked up on gelatine-coated glass slides. Samples were stored at −20 °C.

To measure the myelinated area, sections were stained with LFB (Sigma-Aldrich). Briefly, after a graded dehydration, sections were placed in 1 mg/mL of LFB solution in 96% EtOH and 0.05% acetic acid overnight at 37 °C and protected from light. Then, slides were washed with 96% EtOH, rehydrated in distilled water and placed in a 0.5 mg/mL Li_2_CO_3_ solution for 3–5 min at room temperature. Finally, sections were washed in distilled water, dehydrated again in 100% EtOH and mounted in DPX (Sigma-Aldrich). To assess the demyelinated area in the spinal cord, 6 random images per mouse were captured at 10X magnification with an Olympus BX51 and the attached Olympus DP73 Camera. Demyelination was assessed by measuring the demyelinated areas within the spinal cord white matter. These analyses were done using the Image J analysis software.

To analyse axonal damaged, sections were incubated overnight at 4ºC with an antibody against neurofilament (1:1000; Millipore). Next, sections were washed twice in phosphate-buffered saline (PBS) and incubated for 1 h at room temperature with the secondary antibody donkey anti-rabbit Alexa Fluor 594 (1:500; Invitrogen) and washed again in PBS and PB. Finally, sections were stained with Dapi (1:1000; Sigma-Aldrich) to label nuclei and mounted in DPX (Sigma-Aldrich) after a graded dehydration. To analyse the samples, random images were captured at 40X magnification with an Olympus BX51 and the attached Olympus DP73 Camera.

### Cytokine expression

EAE mice were euthanized at peak of the disease (17 days after EAE induction) with an overdose of pentobarbital sodium (Dolethal) and transcardially perfused with 60 mL of sterile saline (0.9% NaCl). Spinal cords were harvested and rapidly frozen in liquid nitrogen. Protein isolation from the spinal cord samples and cytokine quantification were performed as described previously [28]. The protein levels of the following cytokines (IL-4, IL-10, IL-1α, IL-1β, IL-3, IL-6, TNF-α, IFN-γ, IL-17A, CSF-3, CCL-5, CCL-2, CXCL-2 and CXCL-10) were analysed using a custom-designed Cytokine Magnetic Bead Panel (Invitrogen) on a MAGPIX system (Millipore).

### Flow cytometry

Immune cell infiltration was determined from blood, lymph nodes and spinal cord of EAE mice at the peak of EAE (17 days after EAE induction). Briefly, mice were euthanized with an overdose of pentobarbital sodium (Dolethal). 15 µL of blood was collected from a cardiac puncture and stored in heparinized vials at 4ºC. Then, mice were transcardially perfused with 60 mL of sterile saline (0.9% NaCl) and the spinal cords and lymph nodes (cervical and inguinal) were collected.

Blood samples were incubated with red blood cell lysis buffer (BioLegend) according to manufactures’ guide to obtain a cell suspension enriched in leukocytes. Spinal cords and lymph nodes were cut into small pieces and enzymatically dissociated in 1 mL of Hank’s Balanced Salt Solution (HBSS; Sigma-Aldrich) without Ca^2+^/Mg^2+^ containing 0.1% collagenase and 0.1% DNase for 30 min at 37 °C, and then, mechanically disintegrated through a 70-µm cell strainer to obtain a cell suspension [28].

Cell suspensions were split into different 1.5 mL microcentrifuge tubes according to the number of antibody combinations. For extracellular staining, samples were split, and unstained cells and isotype-matched control samples were generated to control for nonspecific binding of antibodies and for autofluorescence. For surface staining the following antibodies from eBioscience were used at a 1:300 concentration: CD45-PerCP, CD11b-PE or PE-Cy7, F4/80-PE or -APC, Ly6C-FITC, Ly6G-PE, CD3-FITC-APC-PerCP; CD4-APC-Cy7, CD8-APC, CD49b-PE, CD24-PE, CD16/32 PE, CD206 FITC, CD11a-PE, CD49d-FITC, ICAM-1-FITC and CD62L-PE-Cy7. Samples were incubated with the primary antibodies for 1 h at 4 °C with gentle agitation, washed with Dulbeccos’ Modified Eagle Medium (DMEM)-10% foetal bovine serum (FBS) and centrifuged twice at 300 g for 10 min at 4 °C to remove debris and then fixed with 1% PFA.

For intracellular staining in those samples where required, the following antibodies were also used at 1:300 concentration: FoxP3- PE-Cy7, tBet-PerCP, RORγ-APC, GATA3-PE, IFNy-Alexa488, IL-17A-Alexa488, IL-4-APC, IL-10-Alexa488 or APC (all of them from eBioscience) iNOS-Alexa 647 (Abcam) and Arg1-Alexa 488 (Santa Cruz). Cells were fixed and permeabilized with FoxP3 Transcription Factor Staining Buffer Set (eBioscience). Samples were immunostained with the intracellular antibodies for overnight at 4ºC. In the case cytokines antibodies, samples were incubated 4 h at 37 °C in agitation with Cell Stimulation Cocktail 1X (eBioscience) and Brefeldin A 10 µL/ml (Biolegend) before the fixation and permeabilization for intracellular staining. Finally, stained cells were washed with PBS twice and fixed with 1% PFA. Samples were analysed on a FACS Canto Flow Cytometer (BD Bioscience) and all data were processed using FlowJo® software V.10. Microglia cells were defined as CD45^low^ and CD11b^+^, whereas macrophages were defined as CD45^high^, CD11b^+^ and F4/80^+^ and granulocytes (mainly neutrophils) as CD45^high^, CD11b^+^ and F4/80^−^ according to previous publications [4, 27]

### Statistical analyses

Data are shown as mean ± standard error of the mean (SEM). The Kolmogorov–Smirnov test was used to check whether the data was normal distributed. Two-tailed Student’s t test was used for the comparison between two different groups (qPCR, histological analysis, normalized cytokine levels and flow cytometry analysis). EAE clinical score was analysed by using two-way repeated measures ANOVA with post hoc Bonferroni’s test for multiple comparisons. Cytokine levels expressed in picograms/grams of protein in four different groups were analysed by using a one-way ANOVA with post hoc Tukey’s test for multiple comparison. Differences were considered significant at *p* < 0.05.

## Results

### There is an imbalance between pro-inflammatory and pro-resolving lipid mediators in EAE mice

**Fig. 1:**
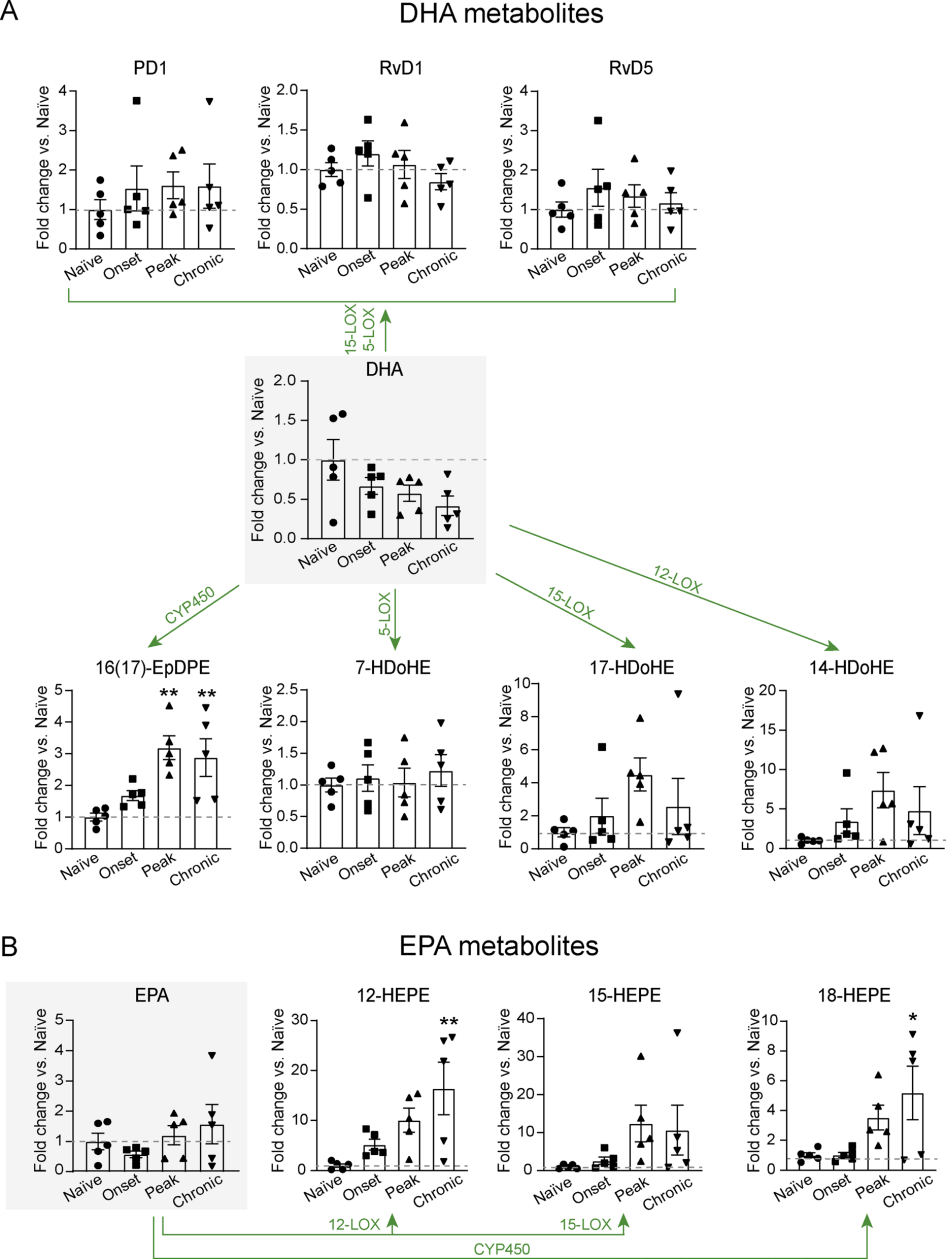
Lipidomic analysis of SPM synthesis pathways markers in the CNS of naïve and EAE mice. **A**, **B** Graphs showing the quantification of **A** DHA metabolome and **B** EPA metabolome in the spinal of naïve and EAE mice (*n* = 5 per group). Dotted line highlights the naïve value. *p < 0.05; **p < 0.01; vs. vehicle. Two-way ANOVA with repeated measures, Bonferroni’s post hoc test. Data are shown as mean ± SEM

We first conducted metabolomic analysis in spinal cord samples of EAE mice to study whether the production of SPMs was induced in the CNS of one of the most widely used animal models of MS. We found that levels of DHA dropped in the spinal cord of EAE, especially at chronic stages of the disease, although this reduction did not reach statistical significance (Fig. 1A).

Further analysis of DHA metabolites revealed some DHA mediators produced by 12/15LOX, such as 14HDoHA and 17HDoHA, which are pathway markers for the formation of Maresins, RvD series and Protectin D1 (PD1), tended to increase at these EAE stages, but not at the significant level (Fig. 1A). Although PD1, RvD1 and RvD5 were detected in the spinal cord at physiological conditions, their levels did not increase in any of the EAE stages analysed (Fig. 1A). Other SPMs derived from DHA, such as MaR1, MaR2 and some members from the RvD series, such as RvD2, Rv3 and Rv4, were undetected in the mouse spinal cord at physiological conditions and during EAE disease. The only DHA metabolite that was significantly augmented in EAE mice was the 16(17)-EpDPE, which is produced via cytochrome P450 (CYP450) and has unknown functions (Fig. 1A).

Levels of EPA remained altered in the spinal cord of EAE mice, although they tended to be higher at chronic stages of the disease (Fig. 1B). However, lipid mediators generated from EPA by 12/15LOX, such as 12-HEPE and 15-HEPE, were increased in the spinal cord of EAE, especially at the chronic phase of the disease, but only 12-HEPE levels reached statistical significance (Fig. 1B). The levels of 18-HEPE, the RvE-series lipid intermediate produced via CYP450, were significantly increased in the spinal cord of chronic EAE (Fig. 1B). However, none of the members of the RvE series were detected.

**Fig. 2:**
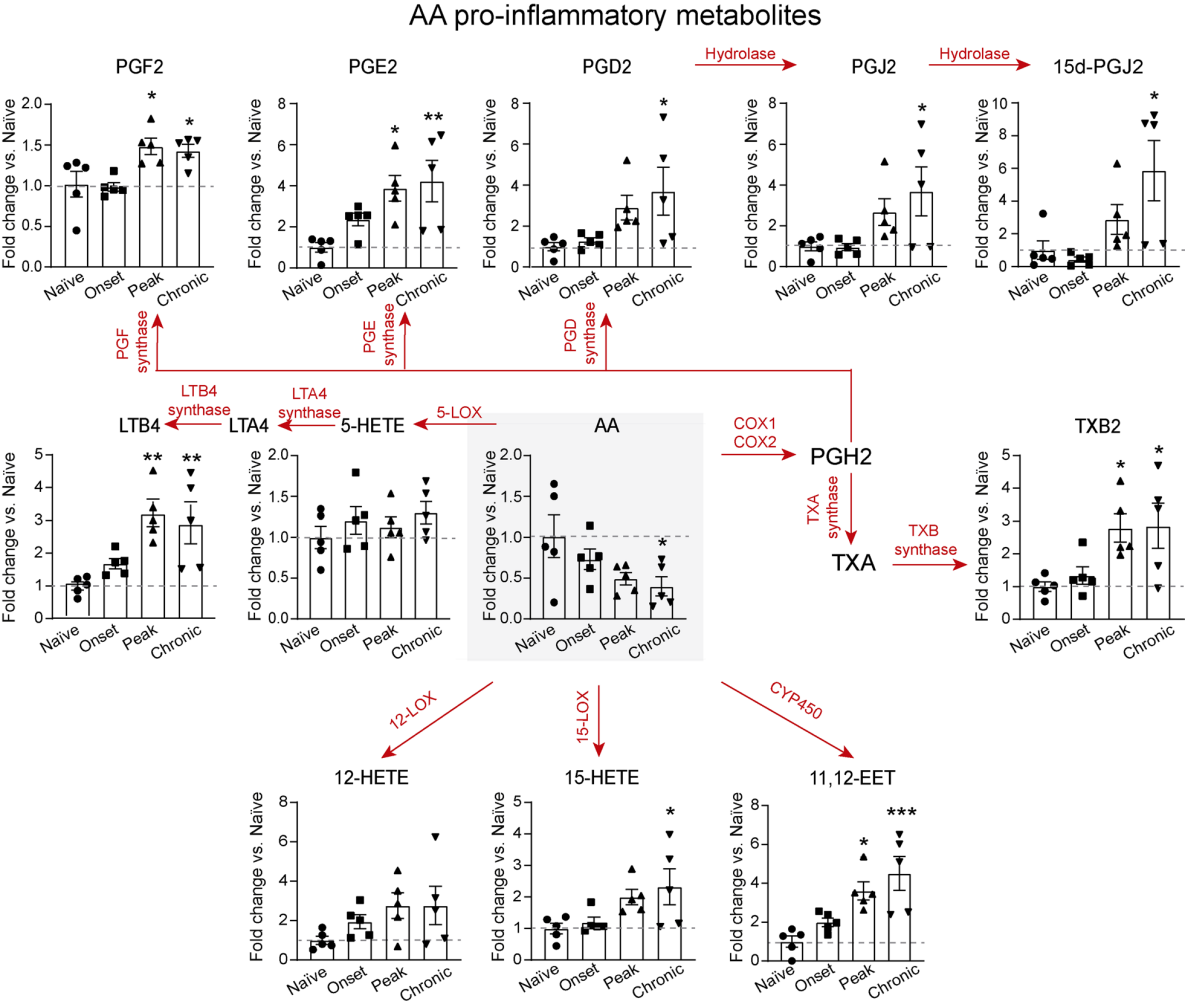
Lipidomic analysis of arachidonic acid pro-inflammatory metabolites in the CNS of EAE and naïve mice. Graphs showing the levels of lipid mediators of the AA metabolome in the spinal of C57Bl/6 mice in physiological conditions and at different stages of EAE (*n* = 5 per group). Red arrows and text indicate the pro-inflammatory metabolite conversion and its implicated enzyme. Dotted line highlights the naïve value. *p < 0.05; **p < 0.01; ***p < 0.001 vs. vehicle. Two-way ANOVA with repeated measures, Bonferroni’s post hoc test. Data are shown as mean ± SEM

**Fig. 3:**
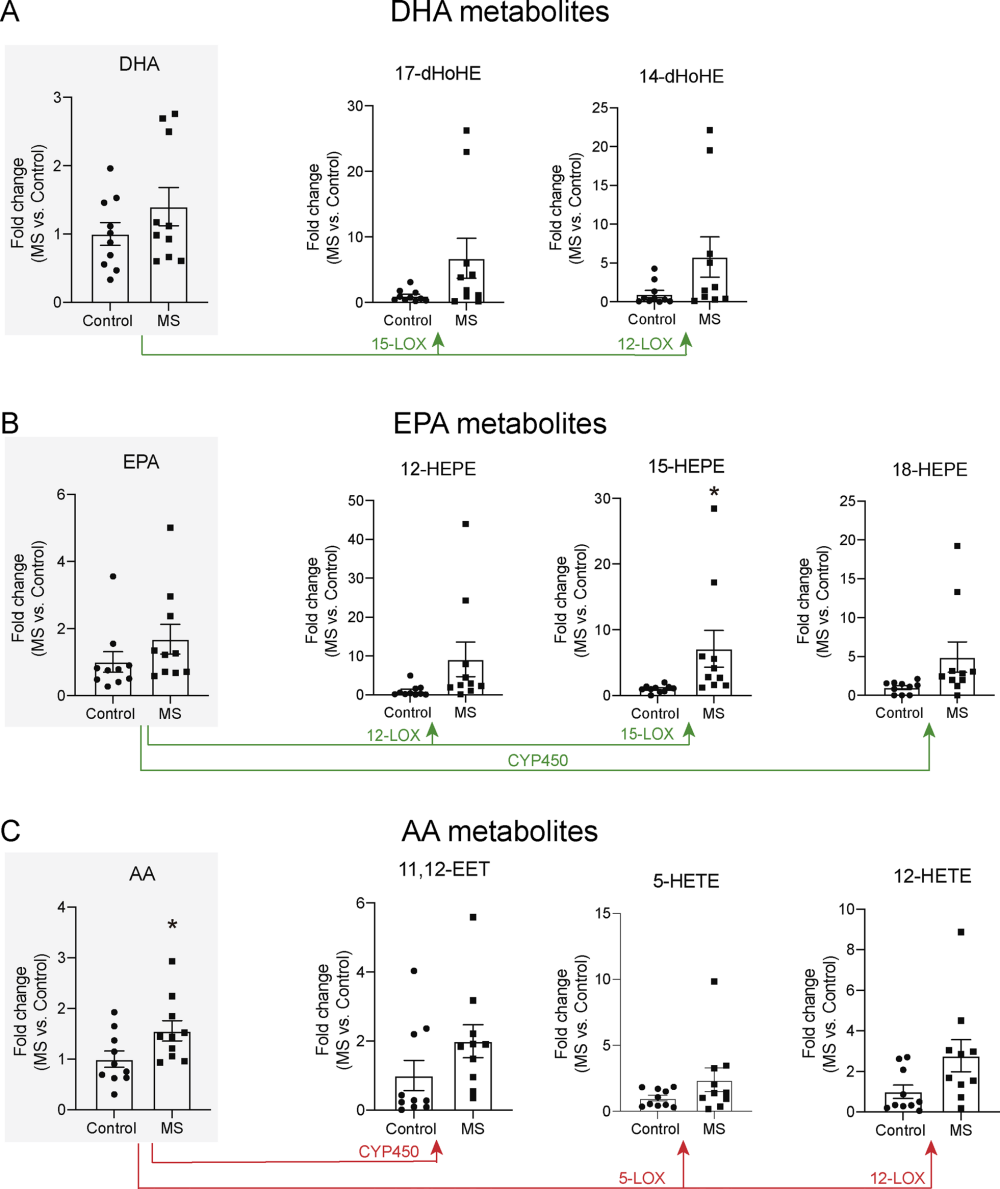
Lipidomic analysis of SPM synthesis pathways markers in serum of healthy controls and MS patients.** A**–**C** Graphs showing the quantification of **A** DHA metabolome, **B** EPA metabolome **C** AA metabolome from serum samples of healthy volunteers (*n* = 10) and MS patients (*n* = 10). *p < 0.05 vs. Control. Unpaired t-test. Data are shown as mean ± SEM

As for the AA metabolome, we found that the levels of the precursor (AA) were reduced in the spinal cord of EAE mice, reaching statistical significance at chronic stages of the disease (Fig. 2A). However, we found a significant increase in the levels of 11,12-EET, which is generated by CYP450, at the peak and chronic phase of EAE disease (Fig. 3C). Lipid mediators generated from AA by 12/15LOX, such as 12-HETE and the intermediate metabolite in LXA4 and LXB4 synthesis, 15-HETE, were also increased in the spinal cord of EAE mice. However, significant differences were only observed in 15-HETE levels at chronic stages of the disease (Fig. 2), while LXA4 and LXB4 were undetected.

In contrast to SPMs, most of the pro-inflammatory lipid mediators derived from AA, such as LTB4, PGD2, PGE2, PGF2 and TXB2, were significantly increased in the spinal cord of EAE mice at the peak and/or chronic stage (Fig. 2).

These data suggest there is an imbalance between pro-inflammatory and pro-resolving lipid mediators in the spinal cord of EAE mice, in favour of the former,

### Synthesis of SPMs is impaired in MS patients

We then performed metabolomic analysis on serum and brain samples of MS patients. These studies revealed that the serum levels of DHA, EPA and AA did not change between MS patients and healthy controls (Fig. 3A–C). Analysing the lipid mediators derived from the DHA metabolome in serum samples, we found that 14-HDoHE and 17-HDoHE, the maresin and resolvin D-series pathway markers, were increased in MS patients yet not significant (Fig. 3A). As for the EPA metabolome, the levels the RvE1 metabolic intermediate, 18-HEPE, as well as 12-HEPE and 15-HEPE were higher in MS patients, although only 15-HEPE reached statistically significance (Fig. 3B). Furthermore, as for the AA metabolome, the levels of 11,12-EET, 5-HETE and 12-HETE were also increased in the serum of MS patients yet not significantly (Fig. 3C). Despite we found several SPM intermediate precursors derived from DHA, EPA and AA in the serum samples, SPMs were undetected in serum samples of MS patients and healthy donors.

**Fig. 4:**
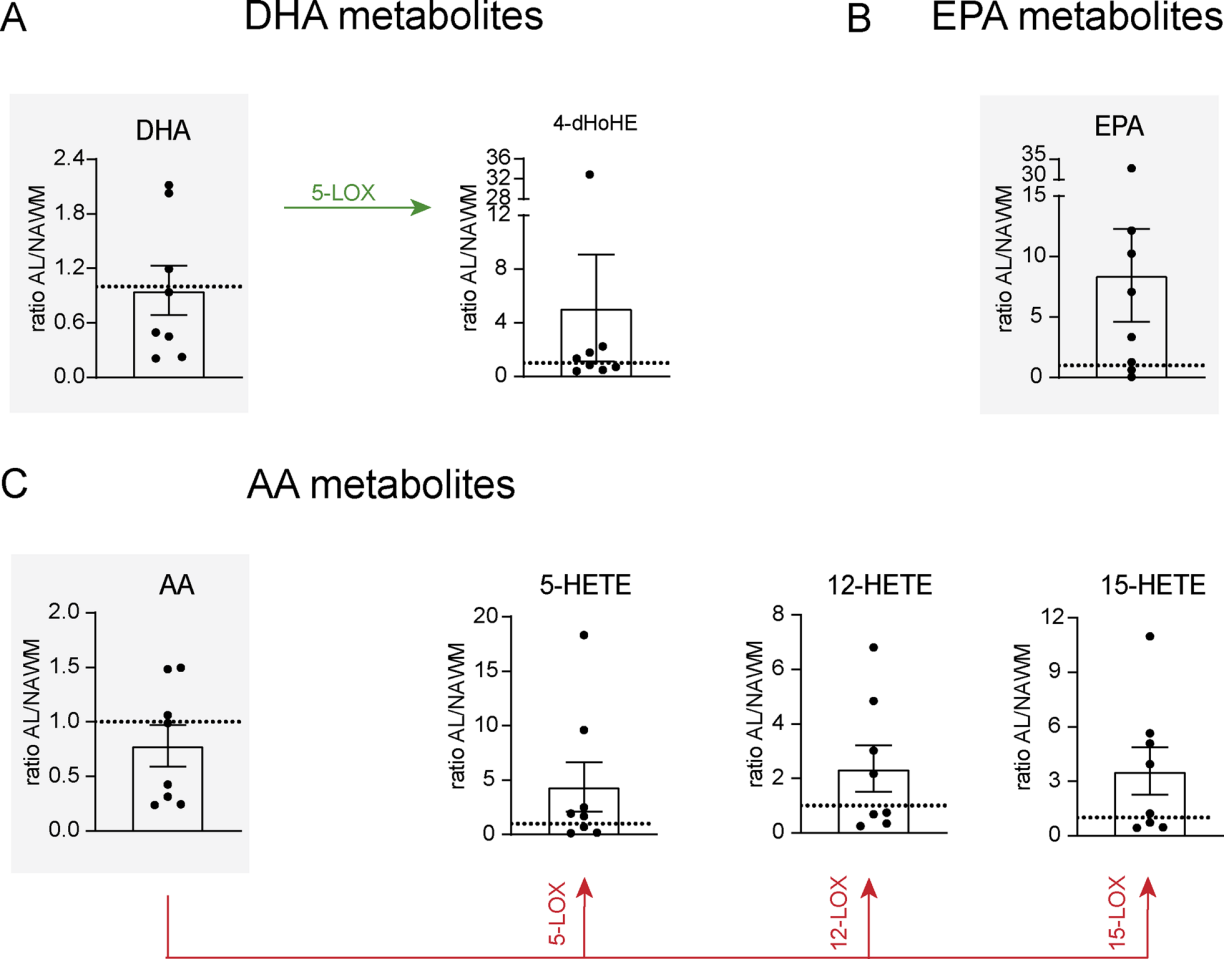
Lipidomic analysis of SPM synthesis pathways markers in brain samples of MS patients.** A–C** Graphs showing the quantification of **A** DHA metabolome, **B** EPA metabolome and **C** AA metabolome in NAWM and AL (*n* = 8). Paired *t*-test. Data are shown as mean ± SEM

We then performed metabolomic studies in active lesions (AL) and normal-appearing white matter (NAWM) of MS post-mortem brain tissue to investigate whether the synthesis of SPMs was defective at the specific regions of the CNS undergoing inflammation. We found that DHA, EPA and AA levels did not vary significantly in AL relative to NAWM, although half of MS individuals showed mark increase in EPA levels in AL (Fig. 4A–C). We also detected some intermediate precursors of SPM synthesis in brain MS samples, although no significant differences were found in AL as compared to NAWM (Fig. 4A–C). From the DHA metabolome, the only lipid mediator detected was the 4-HDoHE, a LOX-5 metabolite that in animal models has been shown to mediate the antiangiogenic effect of omega-3 fatty acids [29]. However, the levels of 4-HDoHE in AL were higher in only 2 out 8 MS patients, but no significant differences were found between AL and NAWM (Fig. 4A). Lipid mediators generated from the EPA metabolome were undetected in AL and NAWM. As for the AA metabolome, we found that 5-HETE, 12-HETE and 15-HETE levels in AL were higher in 4 out of 8 patients, but no significant changes were observed (Fig. 4C). Similar to serum samples, we did not detect any SPM in the MS brain AL and NAWM. Collectively, these data suggest that similar to EAE mice, the class switch from pro-inflammatory to pro-resolution lipid mediators does not occur properly in the periphery and in the CNS of MS patients.

### Feeble expression of *ALOX12* and *ALOX15* in MS patients and EAE mice

**Fig. 5:**
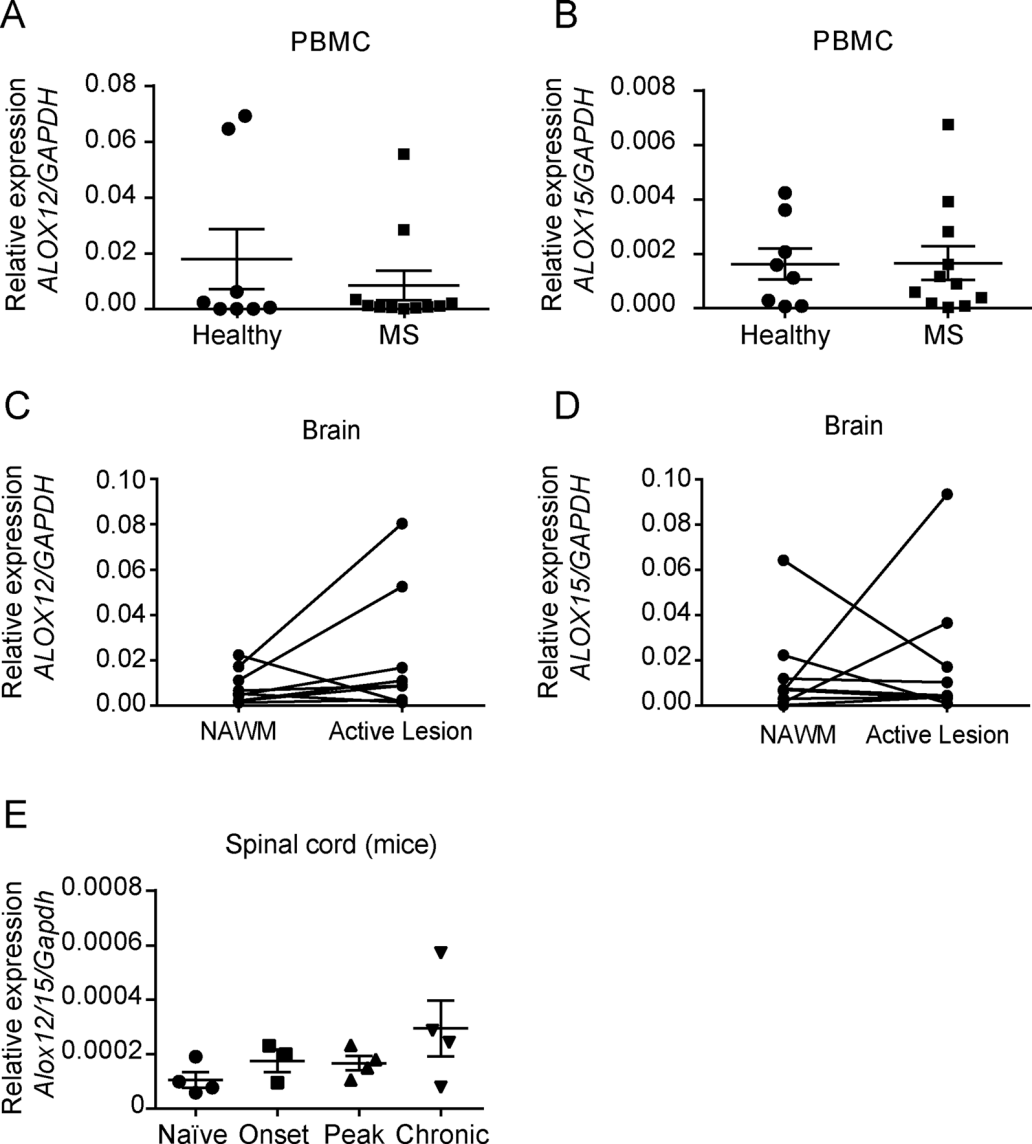
Expression of lipoxygenase 12 and 15 in MS patients and lipoxygenase 12/15 in EAE mice. **A**–**D** Graphs showing the mRNA levels of *ALOX12* **A**, **C** and *ALOX15* **B**,** D** in PBMCs of MS individuals (*n* = 11) and healthy donors (*n* = 8) **B**, **C** and in MS brain AL and NAWM **C**, **D** (*n* = 9). **E** Time course expression of *Alox12/15* mRNA levels in the spinal cord of EAE mice over disease progression (*n* = 3–4 per time point). Unpaired *t*-test in **A**–**D** and one-way ANOVA, *Bonferroni’s *post hoc test in (**E**). Data are shown as mean ± SEM

Since our data suggest there is impaired activation of the resolution programmes of inflammation in MS patients and EAE mice, we then assessed whether this was associated with a failure in the expression of the key biosynthetic enzymes involved in SPM production. Thus, we measured the expression of lipoxygenase-12 (*ALOX12*) and lipoxygenase-15 (*ALOX15*) in PBMCs of active MS patients and healthy donors, as well as in active MS lesions in post-mortem brain tissue. qPCR analysis revealed that mRNA levels of *ALOX12* and *ALOX15* were undetected or found at very low levels PBMCs of either healthy donors or MS patients (Fig. 5A, B). Then, when assessing the expression of these enzymes in post-mortem MS brain samples, we did not find significant changes in the expression within the lesioned areas compared to the NAWM, despite some variability across patients (Fig. 5C, D). Indeed, only 2 out of 9 patients showed increased expression of these enzymes in the AL compared to NAWM, while the rest did not show any change or a slight downregulation (1 patient in *ALOX12* and 2 in *ALOX15*) (Fig. 5C, D).

We then studied whether this phenomenon also occurred in EAE, one of the most widely used animal models of MS. For this purpose, we analysed the expression of *Alox12/15*, the murine orthologue of the previous mentioned enzymes in humans and the initiating enzyme in the SPM biosynthetic pathway. qPCR analysis revealed that, similar to MS patients, mRNA levels of *Alox12/15* were barely found in the CNS of EAE mice, although the transcripts of this enzyme tended to be greater at the chronic stage of the disease (Fig. 5E).

Overall, these results suggest that the key enzymes involved in the production of SPMs are not induced in MS individuals nor EAE mice.

### Effects of MaR1 on inflammation in EAE

**Fig. 6:**
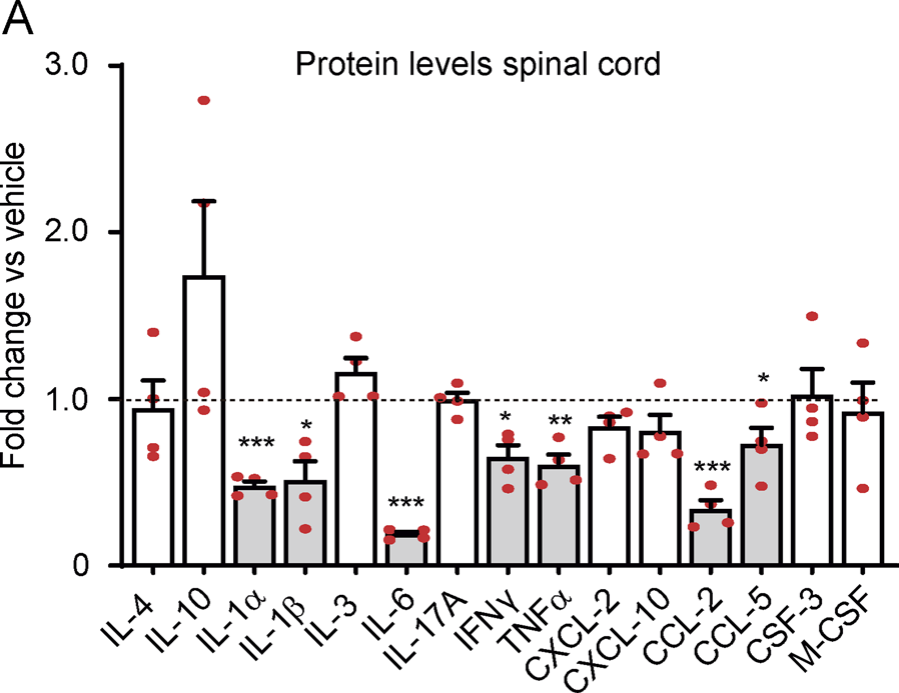
MaR1 reduces pro-inflammatory cytokines in the spinal cord of EAE mice. Plot showing the fold change in cytokine protein levels of different cytokines in the spinal cord of EAE mice treated with MaR1 relative to those treated with vehicle (dotted line). Grey bars highlight those cytokines that are significantly regulated by MaR1. *p < 0.05; **p < 0.01; ***p < 0.001 vs. vehicle. Unpaired *t*-test (*n* = 4 per group). Data are shown as mean ± SEM

Since we demonstrated that production of SPMs is not induced in MS patients and in EAE mice, we proceeded to investigate whether exogenous administration of SPMs attenuates inflammation in EAE. Among the different SPMs, we tested the effects of MaR1 since (i) this SPM was undetected in the serum and brain active lesions of MS patients; (ii) unlikely other SPMs, such as RvD1, RvD5 and NP1, MaR1 was undetected in the spinal cord of EAE mice; (iii) previous studies revealed that this SPM is also undetected in plasma [20] and CFS [6] samples of MS patients; (iv) MaR1, in contrast to other SPMs that were also undetected in MS and EAE patients, such as RvD2–RvD4, has already demonstrated efficacy in minimizing inflammation in the CNS [20]; and (v) the contribution of this SPM in demyelinating diseases is unknown. For this purpose, we administered 1 µg to EAE mice, once a day from disease onset (0.5–1 EAE score) and we assessed the protein levels of 14 cytokines in the spinal cord at the disease peak (4–4.5 EAE score). Luminex assay revealed that MaR1 reduced significantly the levels of 5 out 9 pro-inflammatory cytokines (IL-1α, IL-1β, IL-6, TNFα and INFγ) and 2 out the 4 chemokines measured (CCL-2, CCL-5) (Fig. 6; Additional file 1: Table S3).

**Fig. 7:**
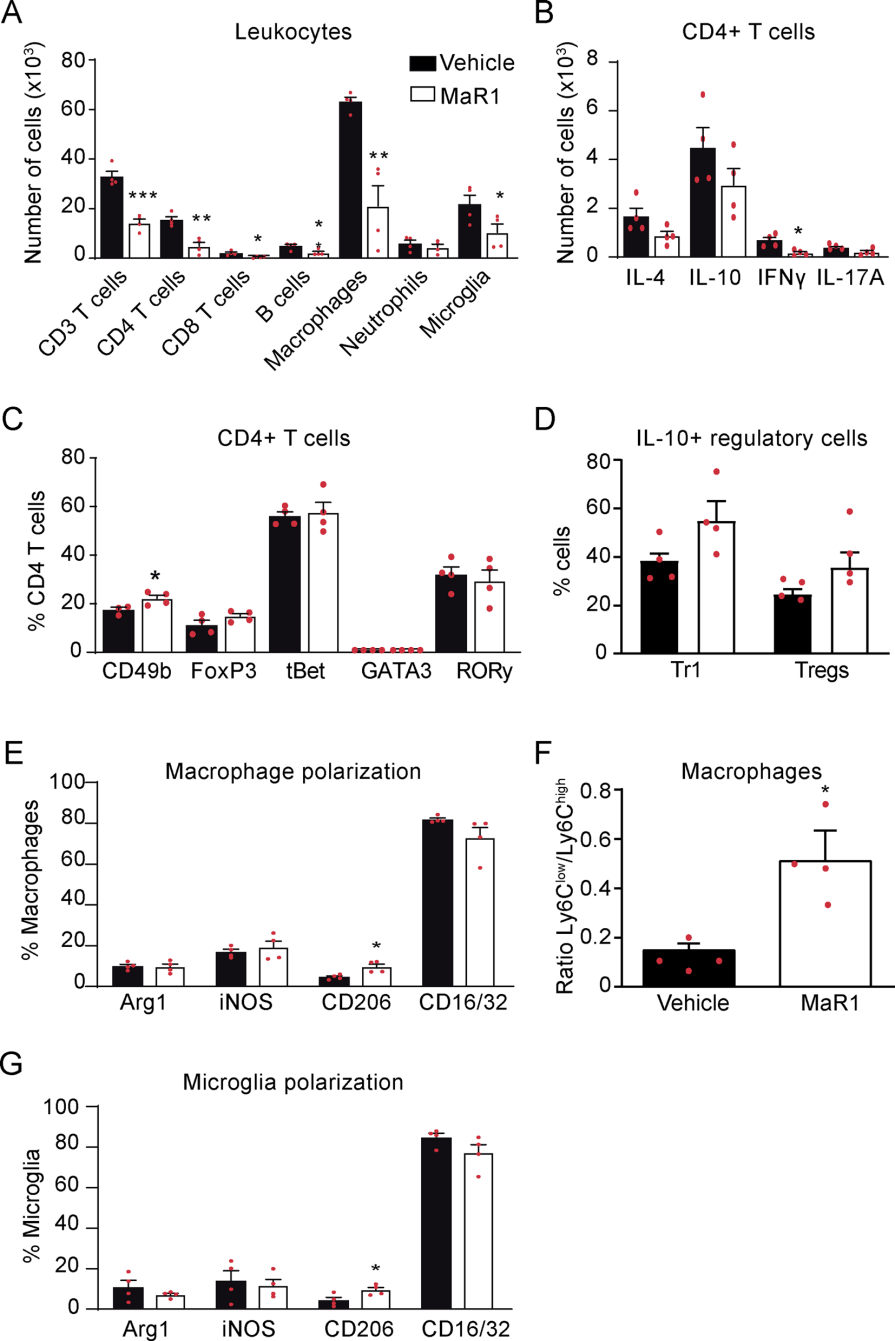
MaR1 attenuates inflammation in the spinal cord of mice undergoing EAE.** A**–**G** Graph showing the quantification of different immune cell populations in the spinal cord (**A**), the number of CD4 + T cells expressing IL-4, IL-10, IFNγ or IL-17A (**B**), the percentage of CD4 + T cells expressing the CD49b, FoxP3, tBet, GATA3 or RORγ **(C),** the percentage of regulatory lymphocytes expressing IL-10 (**D**), the percentage of macrophages expressing polarization markers (**E**), the ratio of Ly6C^low^/Ly6C^high^ macrophages **F** and the percentage of microglial cells expressing polarization markers in the spinal cord after MaR1 or vehicle treatment. *p < 0.05; **p < 0.01; ***p < 0.001 vs. vehicle. Unpaired *t*-test (*n* = 4 per group). Data are shown as mean ± SEM

Since cytokines and chemokines coordinate the migration of immune cells into tissues, we next studied whether MaR1 modulates the number of immune cells in the spinal cord of EAE mice at the peak of the disease. Flow cytometry experiments revealed that MaR1 strongly reduced the numbers of T cells, including both CD4 + T cells (CD3 + , CD4 +) and CD8 + cells (CD3 + , CD8 +), and B cells (CD45 + , CD11b–, CD3–, CD24 +). MaR1 also reduced the accumulation of macrophages (CD45high, CD11b + , F4/80 +) and activated microglial cells (CD45low, CD11b +) but not neutrophils (CD45high, CD11b + , F4/80–, Ly6G +) at this time point (Fig. 7A; Additional file 1: Fig. S1).

Due to the role of cytokines and SPMs in modulating T cell and macrophage phenotypes [4, 30], we investigated whether MaR1 affects these immune cells responses. We found that MaR1 reduced the number of CD4 T cells expressing IFNγ in the spinal cord of EAE mice, suggesting that the number of pathogenic Th1 cells was reduced (Fig. 7B; Additional file 1: Fig. S2B). However, MaR1 did not alter the percentage of T cells expressing tBet, a transcription factor associated with Th1 responses (Fig. 7C). MaR1 did not attenuate the infiltration of Th2 nor Th17 cells since the expression signature cytokines and transcription factors, GATA3 and RORγ, respectively, remained stable after treatment (Fig. 7B, C). However, MaR1 increased the proportion of Tr1 cells (CD3 + , CD4 + , CD49b +) (Fig. 7C; Additional file 1: Fig. S2B), which have a crucial role in maintaining tolerance to self-antigens, and increased in ~ 50% the percentage of this lymphocyte subset producing IL-10, although not to the significant level (Fig. 7D; Additional file 1: Fig. S2C). MaR1 treatment, however, did not alter the percentage of Treg cells (CD3 + , CD4 + , FoxP3 +) (Fig. 7C; Additional file 1: Fig. S2C), another subset of regulatory lymphocytes, but it tended to increase their production of IL-10 (Fig. 6D; Additional file 1: Fig. S2C).

As for macrophages, we found that MaR1 reduced the accumulation of this myeloid cells in the spinal cord of EAE mice, but it increased the percentage of CD206 + macrophages (CD45high, CD11b + , F4/80 + , CD206 +) (Fig. 7A, E; Additional file 1: Fig. S3A), indicating that this SPM increased the proportion of anti-inflammatory macrophages. In this line, we also found that MaR1 bolstered the ratio Ly6C^low^/Ly6C^high^ in macrophages by ~ threefold, further supporting that this SPM favoured the anti-inflammatory responses of macrophages. (Fig. 5F; Additional file 1: Fig. S4B). Similar to macrophages, microglia counts were markedly reduced by MaR1 (Fig. 7A). However, this SPM led to increased proportion of anti-inflammatory CD206 + microglia (CD45low, CD11b + , CD206 +) (Fig. 7G; Additional file 1: Fig. S3C).

We then assessed whether the reduction in leukocyte infiltration in the spinal cord by MaR1 was associated with a reduction in the expression of adhesion molecules in different population of immune cells. We found that MaR1 did not alter the expression of any of the studied adhesion molecules (CD11a, ICAM-1, VLA-4 and CD62L) in macrophages, microglia, granulocytes or lymphocytes (Additional file 1: Fig. S4A–C), suggesting that the reduced infiltration of leukocytes into the CNS of EAE mice treated with MaR1 was likely related to the reduced expression of pro-inflammatory cytokines in the spinal cord rather than to an effect of MaR1 on leukocyte adhesion on blood vessels. Nonetheless, we did find an increase in the percentage of macrophages expressing VLA-4 (CD45high, CD11b + , F4/80 + , VLA-4 +) in MaR1-treated mice (Additional file 1: Fig. S4A), possibly associated with the enhanced infiltration of anti-inflammatory macrophages that we described before.

**Fig. 8:**
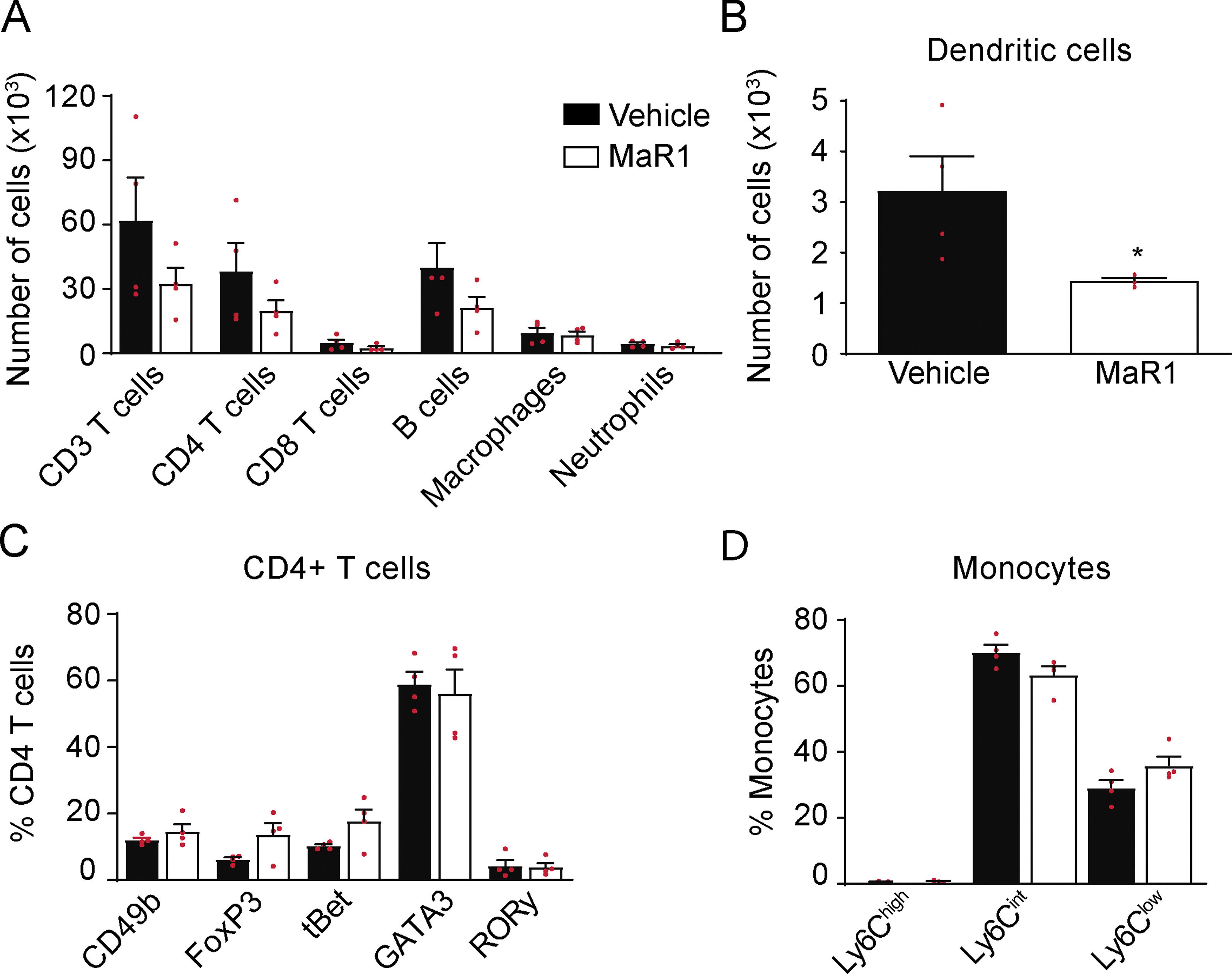
Effects of MaR1 on immune cells in the lymph nodes at the peak of EAE. **A** Graph showing the quantification of different leukocytes populations and dendritic cells **B** in the lymph nodes of MaR1- or vehicle-treated mice. **C** Plot showing the percentage of CD4 + T cells expressing the transcription factors CD49b, FoxP3, tBet, GATA3 or RORγ and the cell numbers for the different macrophage subsets according to Ly6C expression **D** in the lymph nodes of MaR1- or vehicle-treated mice. Unpaired *t*-test (*n* = 4 per group). Data are shown as mean ± SEM

Next, we investigated whether the lessened leukocyte infiltration in the CNS after MaR1 treatment is linked with the attenuation of immune cells expansion in the lymph nodes or in the blood stream. We found that MaR1 slightly reduced the number of the different leukocyte populations in the lymph nodes, although the result was not statistically significant (Fig. 8A). However, MaR1 reduced by ~ twofold the number of dendritic cells (CD45high, CD11b + , F4/80 + , CD11c), which have a decisive role in T cell priming. However, in contrast to that observed in the spinal cord, MaR1 did not modulate the phenotype of CD4 T cells and monocytes. (Fig. 8C–D).

**Fig. 9:**
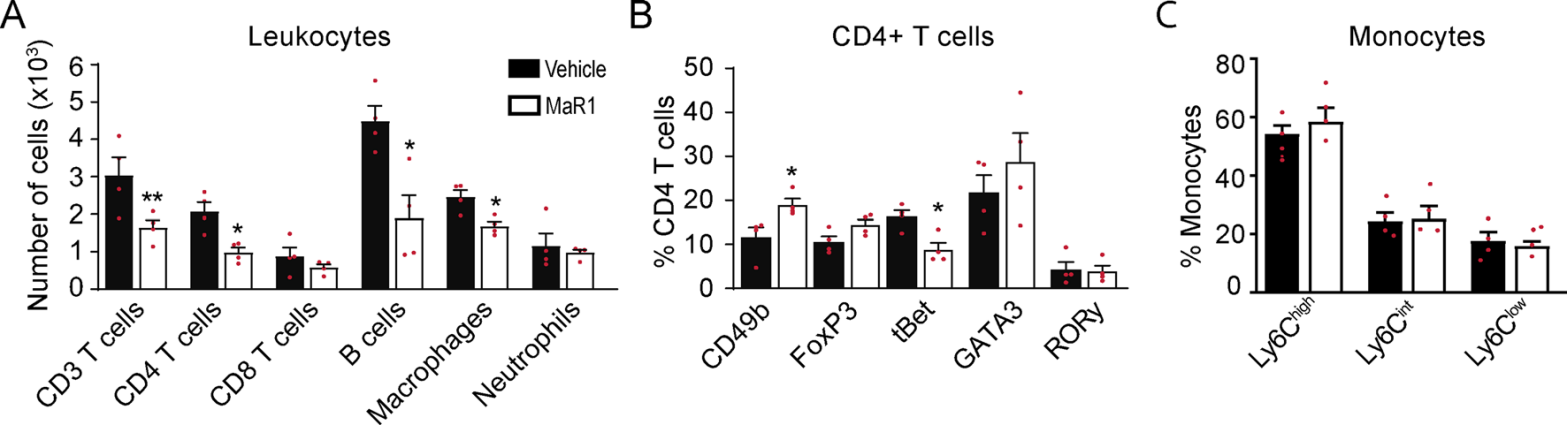
Effects of MaR1 on immune cells in the blood of EAE mice at disease peak. **A** Graph showing the number of different leukocytes populations in the blood of MaR1- or vehicle-treated mice. **B** Plot showing the percentages of CD4 + T cells expressing the transcription factors CD49b, FoxP3, tBet, GATA3 or RORγ and **D** different macrophage phenotypes according to Ly6C expression in blood of EAE mice treated with MaR1 or vehicle. Unpaired *t*-test (*n* = 4 per group). *p < 0.05; **p < 0.01 vs. vehicle. Unpaired *t*-test (*n* = 4 per group). Data are shown as mean ± SEM

Despite the minimal actions of MaR1 on lymphocyte and monocyte in the lymph nodes, we observed fewer CD4 T cells, B cells and monocytes in the blood of EAE mice (Fig. 9A). MaR1 also led to changes in the phenotype of circulating CD4 T cells, increasing the percentage of Tr1 cells and also reducing that of pathogenic Th1 cells (Fig. 9B). Nonetheless, MaR1 did not modify the phenotype of circulating monocytes, which had a predominant pro-inflammatory state in both treated and no treated mice (CD45high, CD11b + , F4/80 + , Ly6C^high^) (Fig. 9C). These results suggested that some immune cell responses observed in the spinal cord of MaR1-treated mice might be, in part, due to the actions of this SPM in the periphery.

### Systemic administration of MaR1 attenuates EAE

**Fig. 10:**
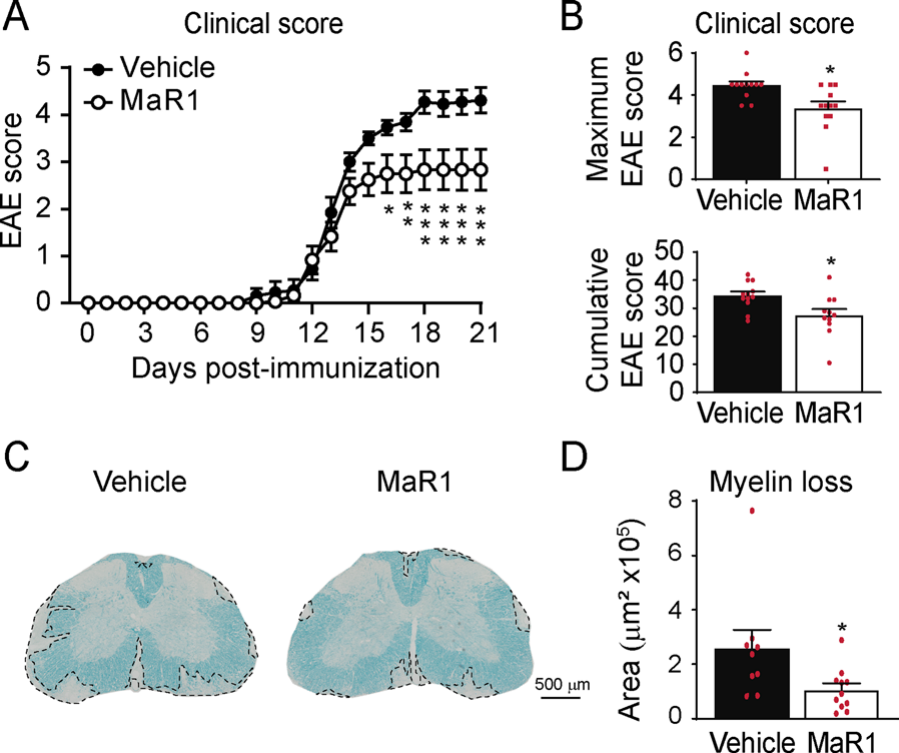
Effects of MaR1 on neurological deficits and myelin loss in EAE mice. **A**, **B** Graphs showing the clinical score of EAE mice treated with MaR1 or vehicle over disease progression (**A**), as well as the maximum and cumulative EAE score (**B**).** C** Graph showing the quantification of myelin loss in the lumbar spinal cord of MaR1- or vehicle-treated mice at 21 days post-induction. **D** Representative histological spinal cord tissue sections stained with LFB from EAE mice treated with vehicle and MaR1. *p < 0.05; **p < 0.01; ***p < 0.001 vs. vehicle. Two-way ANOVA with repeated measures, Bonferroni’s post hoc test in A (*n* = 12 per group). Unpaired t-test in B (*n* = 12 per group) and C (*n* = 9 in vehicle and *n* = 11 in MaR1). Data were pooled from two different experiments. Data are shown as mean ± SEM

To investigate whether the effects of MaR1 on inflammatory processes carry a clinical outcome, we assessed the effects of exogenous delivery of MaR1 on EAE symptoms. We found that mice treated with MaR1 were markedly protected against neurological impairments, despite the treatment being initiated at disease onset (data pooled from two independent experiments) (Fig. 10A, B). Indeed, MaR1-treated mice reached an average EAE score of ~ 2.5 by the end of the follow-up, while vehicle-treated mice reached ~ 4.2 (Fig. 10A). Moreover, mice treated with MaR1 had significantly lower cumulative and maximum EAE scores than vehicle-treated mice (Fig. 10B).

We then evaluated whether the enhancement in neurological functions observed in EAE mice treated with MaR1 was related to preservation of myelin within the CNS. (Fig. 10C, D). However, histological sections immunostained against NF revealed that there were very few axons within the demyelinating lesions of mice treated with MaR1 or vehicle (Additional file 1: Fig. S5). These data suggest that the reduced neurological deficits observed in EAE mice treated with MaR1 was due to the ability of this SPM to reduce the size of the CNS lesions rather than to remyelination.

Overall, these data reveal the therapeutic potential of MaR1 in attenuating neuroinflammation and protecting from tissue damage and clinical signs of EAE.

## Discussion

In the present study, we report that the production of the main lipid mediators that activate the resolution programmes of inflammation is deficient in serum and active brain lesions of MS patients, as well as, in the CNS of EAE mice. These findings are associated with impaired induction of key enzymes involved in the biosynthetic pathways of SPMs. We also reveal that the administration of the resolution agonist, coined MaR1, in EAE mice attenuates the pro-inflammatory environment of the CNS, modulates T cell and macrophage responses towards a more regulatory or anti-inflammatory phenotype, and reduces the blood counts of different leukocyte populations. Importantly, we demonstrate that the effects of MaR1 on inflammation are associated with marked protection against neurological decline and myelin loss despite treatment initiation at disease onset.

PUFAs play a critical role in the regulation of inflammation by controlling several processes involved in its onset and termination [2, 31, 32]. In general, eicosanoids generated via the actions of cyclooxygenase 1 and 2 (COX-1 and 2) and 5-LOX from AA, such as prostaglandins, thromboxanes and leukotrienes, contribute to inflammation by promoting chemotaxis and activation of immune cells and increasing vascular permeability [33, 34]. Nevertheless, not all the actions of eicosanoid are pro-inflammatory since a number of in vivo studies revealed that 15d-Prostaglandin J2, for instance, attenuates inflammation and neurological decline in EAE and spinal cord injury [35, 36].

In contrast, SPMs are key players in the resolution of inflammation. Except for lipoxins, SPMs are derived from omega-3 PUFA, in particular, from DHA and EPA. DHA can give rise to several resolution agonists, including those of the resolvin D series, protectin D and MaRs. EPA can also yield some SPMs, namely, resolvin E series [2, 7, 9, 11, 37, 38].

Omega-3 PUFAs are enriched in oils derived from fish and algae. Various epidemiological studies have indicated that omega-3 PUFA dietary supplementations are associated with improved clinical outcome of various pathologies, such as metabolic syndrome or cardiovascular diseases [39, 40]. In MS, however, the beneficial actions of omega-3 PUFA supplementation are inconsistent, as one trial failed to show clinical improvement despite the effectively increased omega-3 PUFA levels in patients as a result of the supplementation [41, 42]. Thus, it is very plausible that this lack of efficacy stems from the failure of MS patients to produce adequate amounts of SPMs, rather than from the bioavailability of omega-3 PUFA in their serum.

Previous studies have reported that lipid mediator pathways are dysregulated in various nervous system-related pathologies [4, 6, 19, 20, 43]. For instance, inappropriate SPM production has been observed in other CNS conditions, such as in Alzheimer’s [43] and in mice after spinal cord contusion injury [4]. A recent report has uncovered that most SPMs are absent in plasma samples of MS patients at different stages of the disease, and only RvD1, RvD5 and NP1 could be detected [20]. These observations are in agreement with our findings shown here, although we were unable to detect any SPM in serum or inflammatory regions of the brain. These small differences between both studies could be due to type of sample analysed, and in case of the active brain lesions samples, to the limited amount of tissue available. However, RvD1, RvD5 and NPD1 were the three SPMs that we detected in the spinal cord of mice at physiological conditions, but their levels did not increase at different stages of EAE disease. Interestingly, we found that the impaired production of SPMs in serum plasma of MS patients was associated with deficient induction in the expression of the main SPM biosynthetic enzymes in PBMCs, which is in agreement with the work of Kooij and colleagues [20]. Besides, we also provided evidence indicating that this inefficient induction in *12-* and *15-LOX* expression also occurs in regions of the brain undergoing inflammation in MS patients, as well as in the spinal cord of EAE mice. Therefore, these results suggest that there is a failure in peripheral and central production of SPMs in MS, which may be responsible, in part, of the dysregulated deleterious inflammatory response characteristic of this disease.

Interestingly, Kooij and colleagues observed inverse correlation between the plasma levels of these RvD1, RvD5 and NP1 and clinical severity in MS patients [20], and that LXA4, LXB4, RvD1 and PD1 reduced MS-derived monocyte activation and cytokine production, suggesting that administration of SPM could attenuate inflammation in MS patients. In this line, prophylactic administration of RvD1 in EAE alleviated the clinical signs of disease, suggesting that the exogenous administration of SPMs could revert the pathological signs of neuroinflammation [19].

MaR1 is an understudied member of the SPM superfamily [9, 44]. There are just few studies demonstrating the beneficial actions of this SPM in the CNS. MaR1 not only enhanced inflammatory resolution after spinal cord injury and reduced myelin loss and neurological deficits [4], but also promoted neuroprotection in spinal muscular atrophy [45] and cerebral ischemia [46]. Thus, we complete this scenario since herein we provide data demonstrating that the administration of MaR1 in EAE mice also conferred protection against functional deficits and myelin loss, despite initiating the treatment after disease onset. We also found that MaR1 modulates different inflammatory mechanisms. First, MaR1 reduced the protein levels of different pro-inflammatory cytokines in the spinal cord of EAE mice, including IL-1α, IL-1β, TNFα, IFNγ and IL-6, among others, but did not alter the amounts of anti-inflammatory/immunomodulatory cytokines, such as IL-4 or IL-10. These pro-inflammatory cytokines play a critical role in the development of EAE [47–49], and are associated with neurodegeneration and progression in MS [50, 51]. Second, MaR1 reduced the accumulation of major leukocyte populations in the spinal cord of EAE mice, albeit not neutrophils. This may have occurred, at least partially, due to the decreased levels of cytokines in the spinal cord, and also because of MaR1’s ability to hamper the accumulation of immune cells also into the blood. Third, MaR1 modulated the responses of macrophages and CD4 T cells towards a more anti-inflammatory profile. In particular, MaR1 reduced pathogenic Th1 cells in the spinal cord and increased the number of non-classical regulatory T cells. In agreement with our findings, a previous study revealed that resolution agonists, including MaR1, attenuate the activation of pathogenic Th1 and Th17 cells and their generation from naïve CD4 cells [30]. We did not observe any significant change in the cell number of different Th cell populations in the lymph nodes, suggesting that MaR1 does not regulate the peripheral activation of Th cells in EAE, at least when its administration occurs after disease onset. MaR1, however, reduced Th1 cell number in the blood and increased that of non-classical Tregs. In this study, we also observed that macrophages present in the spinal cord of EAE adopted a more anti-inflammatory phenotype, similar to our previous findings in spinal cord injury [4]. Because this was only observed in the CNS and not also in the lymph nodes or blood, it suggests that the modulation of the inflammatory environment of the CNS by MaR1 is most likely to be the responsible for the effects on macrophages. However, the impact of MaR1 on immune cell numbers is unlikely dependent on the modulation of adhesion molecules in peripheral leukocytes or microglial cells since this SPM fail to reduce their expression. Indeed, MaR1 increased the expression of VLA-4 in macrophages. This cell adhesion has been recently shown to be important for the infiltration of Ly6C^low^ macrophages [52] and may account for the higher infiltration of anti-inflammatory macrophages observed in the CNS of EAE mice treated with MaR1.

## Conclusions

Overall, we provide clear evidence that there is inefficient production of SPMs in the spinal cord of EAE mice as well as in the CNS and periphery of MS patients. Our data indicate that exogenous administration of MaR1 is effective at enhancing multiple mechanisms needed for preventing inflammation in EAE mice and confers protection against neurological decline and myelin loss. These data therefore indicate that SPMs in general, and MaR1 in particular, represent a novel therapeutic avenue for the treatment of MS with promising potential.

## Supplementary Information

**Additional file 1. Fig. S1. **MaR1 reduces the accumulation of immune cells in the spinal cord of mice at the peak of EAE. **Fig. S2. **MaR1 modulates T cells responses in the spinal cord of EAE mice. **Fig. S3. **MaR1 modulates the macrophages and microglia phenotypes towards a more anti-inflammatory phenotype. **Fig. S4. **Effects of the treatment with MaR1 in the expression of adhesion molecules on immune cells in the spinal cord at the peak of EAE. **Fig. S5**. Effects of MaR1 in axonal damage in the lesioned areas of the spinal cord at the peak of EAE. **Table S1.** Descriptive information about human samples of MS patients and healthy controls used for QPCR analysis**. Table S2.** Descriptive information about human samples of MS patients and healthy controls used for lipidomic analysis. **Table S3.** Cytokine expression in the spinal cord of naïve, vehicle- and MaR1-treated mice at the peak of EAE.

## Data Availability

All the data generated or analysed during this study are included in this published article ant its supplementary information files.

## Abbreviations

AA: Arachidonic acid
CFA: Complete Freund´s adjuvant
CNS: Central nervous system
DHA: Docosahexaenoic acid
EAE: Experimental autoimmune encephalomyelitis
EPA: Eicosapentaenoic acid
LOX: Lipoxygenase
LX: Lipoxins
MaR1: Maresin-1
MS: Multiple sclerosis
NAWM: Normal-appearing white matter
PBMCs: Peripheral blood mononuclear cells
PBS: Phosphate-buffered saline
PD/NPD: Protectins/Neuroprotectins
PPMS: Primary-progressive multiple sclerosis
PUFAs: Polyunsaturated fatty acids
qPCR: Real-time Quantitative PCR Assay
RRMS: Relapsing–remitting multiple sclerosis
RvD: Resolvin D
RvE: Resolvin E series
sMRM: Scheduled multiple reaction monitoring
SPMS: Secondary-progressive multiple sclerosis
SPMs: Specialized pro-resolving lipid mediators
