# Supplementary material for: Administration of Maresin-1 ameliorates the physiopathology of experimental autoimmune encephalomyelitis
Source: J Neuroinflammation. 2022 Feb 2;19:27. doi: 10.1186/s12974-022-02386-1 (PMC8808957; doi:10.1186/s12974-022-02386-1)
Supplement: Supplementary file 1 — Additional file 1. Fig. S1. MaR1 reduces the accumulation of immune cells in the spinal cord of mice at the peak of EAE. Fig. S2. MaR1 modulates T cells responses in the spinal cord of EAE mice. Fig. S3. MaR1 modulates the macrophages and microglia phenotypes towards a more anti-inflammatory phenotype. Fig. S4. Effects of the treatment with MaR1 in the expression of adhesion molecules on immune cells in the spinal cord at the peak of EAE. Fig. S5. Effects of MaR1 in axonal damage in the lesioned areas of the spinal cord at the peak of EAE. Table S1. Descriptive information about human samples of MS patients and healthy controls used for QPCR analysis. Table S2. Descriptive information about human samples of MS patients and healthy controls used for lipidomic analysis. Table S3. Cytokine expression in the spinal cord of naïve, vehicle- and MaR1-treated mice at the peak of EAE. [file 12974_2022_2386_MOESM1_ESM.pdf]

Additional file 1

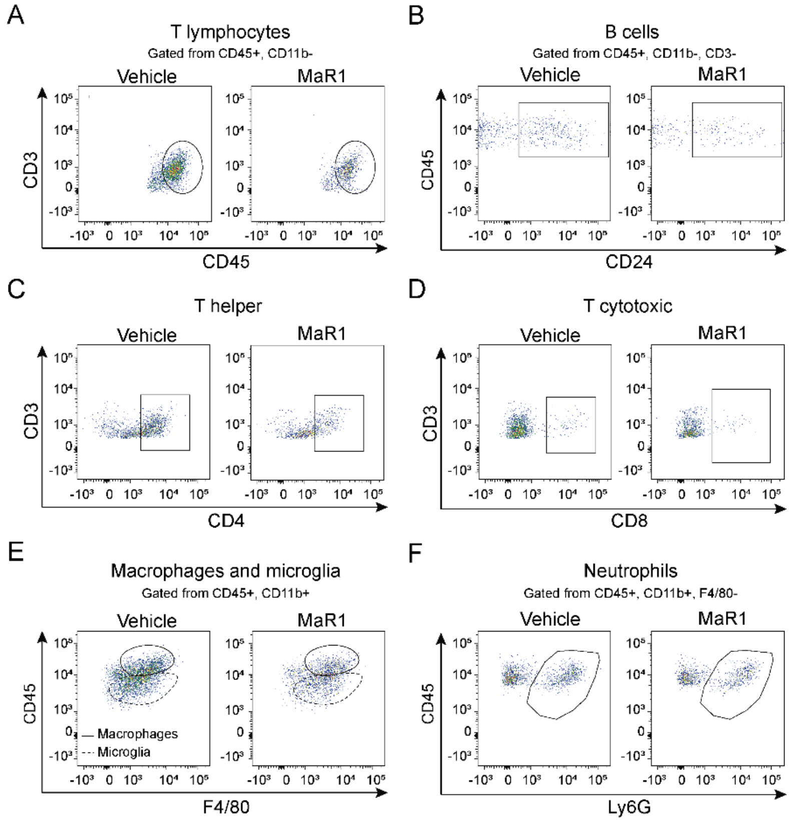

S1. MaR1 reduces the accumulation of immune cells in the spinal cord of mice at the peak of EAE. (A-F) Representative dot plots showing different **(A)** T lymphocytes, **(B)** B cells, **(C)** T helper cells, **(D)** T cytotoxic cells, **(E)** macrophages and microglia and **(F)** neutrophils in the spinal cord at the peak of EAE in vehicle- and MaR1-treated mice.

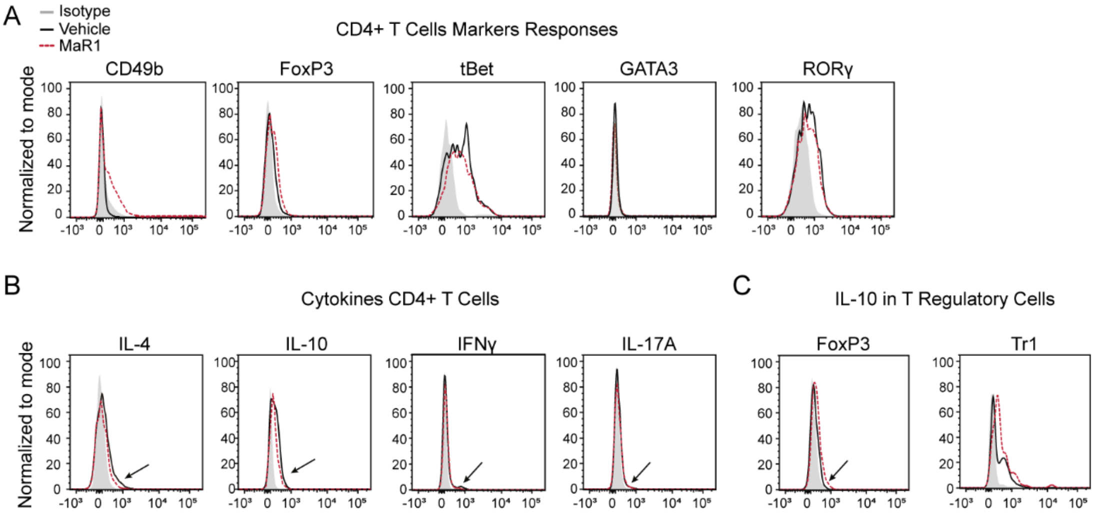

S2. MaR1 modulates T cells responses in the spinal cord of EAE mice. (A-C) Representative flow cytometry histograms showing the expression of different T cell marker responses **(A)**, various cytokines levels in T cells **(B)**, and IL-10 secretion by regulatory lymphocytes **(C)** in the spinal cord at the peak of EAE in vehicle- and MaR1-treated mice.

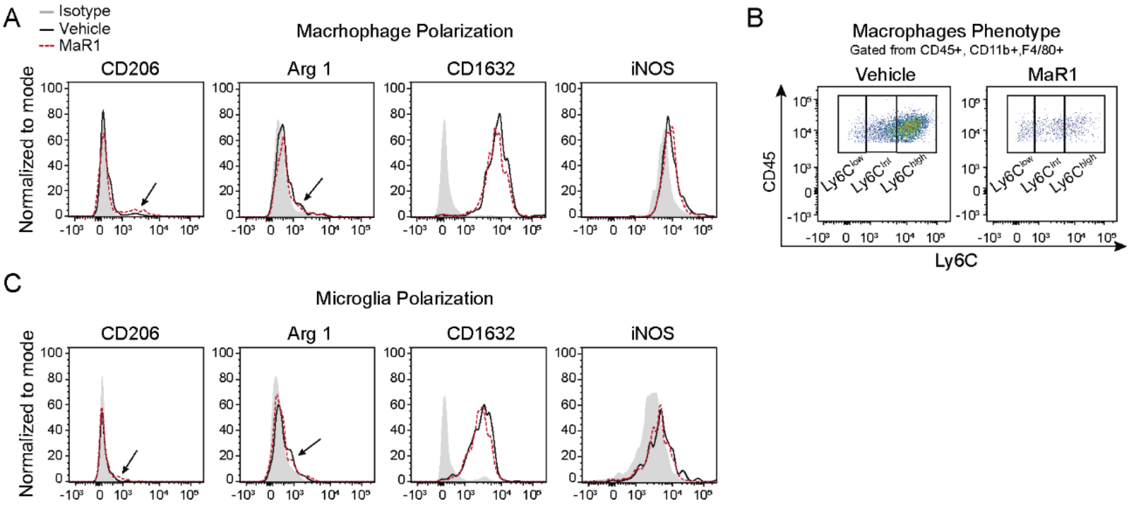

S3. MaR1 modulates the macrophages and microglia phenotypes towards a more anti-inflammatory phenotype. (A-C) Representative flow cytometry histograms showing the expression of M1 markers (CD1632 and iNOS) and M2 markers (CD206 and Arg1) in macrophages **(A)** and microglia **(C)**.Representative dot plots showing the expression of Ly6C^{low}, Ly6yC^{int} and Ly6C^{high} **(B)** in macrophages and M1/M2 markers in microglial cells **(C)** in the spinal cord at the peak of EAE in mice treated with vehicle or MaR1.

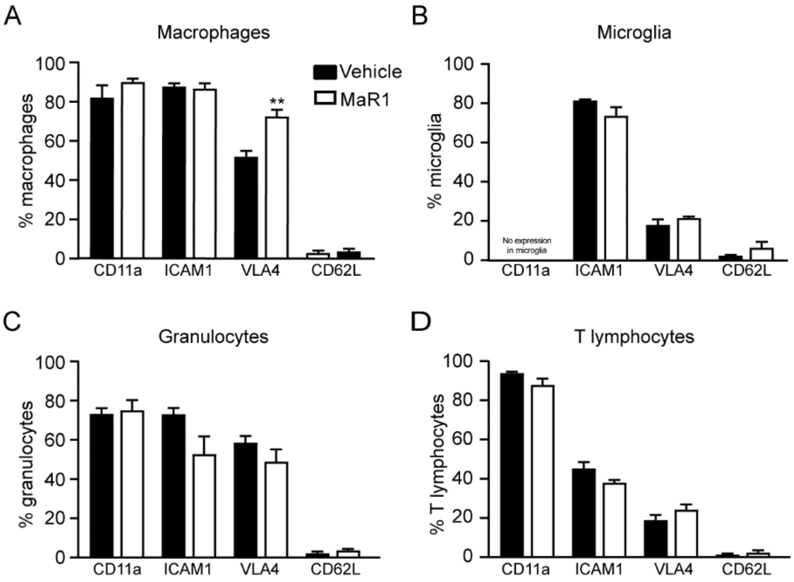

S4. Effects of the treatment with MaR1 in the expression of adhesion molecules on immune cells in the spinal cord at the peak of EAE. (A-D) Graphs showing the percentage of macrophages **(A)**, microglia **(B)**, granulocytes **(C)** and T lymphocytes **(D)** expressing CD11a, ICAM-1, VLA-a or CD62L in the spinal cord of vehicle- or MaR1-treated mice at the peak of EAE.

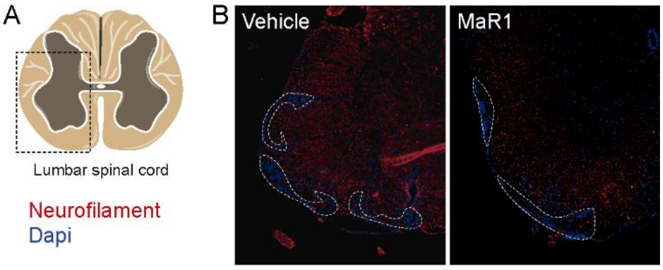

S5. Effects of MaR1 in axonal damage in the lesioned areas of the spinal cord at the peak of EAE. Schematic representation of the lumbar spinal cord **(A)** and representative microphotographs of spinal cord tissue sections immunolabeled against NF200 (red) and cellular nuclei (Dapi; in blue) showing very few axons within the lesions of vehicle- or MaR1-treated EAE mice at day 21 post-induction **(B)**.
